# Expression of GCRG213p, LINE-1 endonuclease variant, significantly different in gastric complete and incomplete intestinal metaplasia

**DOI:** 10.1186/s13000-019-0838-9

**Published:** 2019-06-21

**Authors:** Xiaojian Duan, Hongwei Lian, Jie Li, Benyan Wu, Weihua Wang, Tao Wu, Changzheng Wang, Yan Dou, Zhongren Zhou, Bingzhi Wang, Liyan Xue, Gangshi Wang

**Affiliations:** 1 Department of Gastroenterology, The Second Medical Center, Chinese PLA General Hospital, National Clinical Research Center for Geriatric Diseases, Beijing, 100853 China; 2 Department of Pathology, The First Medical Center, Chinese PLA General Hospital, Beijing, 100853 China; 3 Department of Gastroenterology, The First Medical Center, Chinese PLA General Hospital and National Clinical Research Center for Geriatric Diseases, Beijing, 100853 China; 4 Department of Pathology and Immunology, Washington University School of Medicine, St. Louis, MO 63110 USA; 5 Department of Pathology and Resident Training Base, Cancer Hospital, Chinese Academy of Medical Sciences, Peking Union Medical College, Beijing, 100021 China

**Keywords:** Gastric intestinal metaplasia subtypes, Gastric adenocarcinoma, GCRG213, Chief cells

## Abstract

**Background:**

Intestinal metaplasia (IM) of the gastric mucosa is classified as complete (Type I) and incomplete IM (Type II and III) subtypes, which showed significantly different risk for developing to gastric adenocarcinoma (GAC). GCRG213, a variant of L1-endonuclease (L1-EN), first identified in our lab, was upregulated in GAC tissue. However, the relationship between GCRG213 and IM subtypes is not clear. Our study explored the association of GCRG213 protein (GCRG213p) with IM subtypes.

**Methods:**

Gastric cancer and/or para-tumor tissue samples were collected from 123 patients who underwent gastrectomy for intestinal type gastric adenocarcinoma. The subtypes of IM were characterized with Alcian blue-periodic acid-Schiff and High Iron Diamine-Alcian blue staining methods. Immunohistochemistry of GCRG213p was performed, and its expression in gastric adenocarcinoma and para-tumor tissue including dysplasia, IM, and normal mucosa were analyzed.

**Results:**

GCRG213p was expressed in 48.94% IM, 57.14% dysplasia and 55.32% GAC, respectively. GCRG213p expression was higher in well and moderately differentiated adenocarcinoma (*P* = 0.037). In IM glands, GCRG213p expressed mainly in the cytoplasm of absorptive enterocytes with defined brush borders, but not in goblet cells. The expression of GCRG213p in type I IM (90.00%) was significantly higher than that in type II (36.36%) and type III (25.00%) (*P* < 0.001). In normal gastric mucosa, GCRG213p was exclusively positive in the cytoplasm of gastric chief cells.

**Conclusions:**

The expression of GCRG213p in complete IM was significantly higher than in incomplete IM, which implies that GCRG213p may play a role on the developing of IM to adenocarcinoma. GCRG213p was exclusively expressed in chief cells, suggesting that it might be involved in cell differentiation from the chief cells to IM.

## Background

Gastric cancer is the second highest cause of cancer-related death in the world [1]. The accepted model for the development of the intestinal type of non-cardia gastric cancer, known as Correa’s cascade, consists stepwise progression from chronic active gastritis, multifocal atrophic gastritis, intestinal metaplasia (IM), dysplasia and finally gastric adenocarcinoma (GAC) [2]. IM in the gastric mucosa is a precancerous lesion of GAC and the “Point of no return” [3], which remains the focus of GAC surveillance and prevention [4, 5]. According to the evidence in a nationwide cohort study in Netherlands, the annual incidence of gastric cancer was 0.25% for patients with IM within 5 years after diagnosis [6]. IM also takes a long time to develop to GAC. A recent nationwide observational cohort study in Sweden where the incidence of GAC is relatively low, revealed that approximately 1 in 39 subjects with IM will develop to GAC within 20 years after IM diagnosis [7]. Since the incidence of IM progress to gastric cancer is low, it is not efficient to surveil IM patients for GAC. Currently, the clinical management of the patients with IM still remains of challenging task.

IM has long been recognized as heterogeneous, which is classified into three subtypes based on mucin stain, including type I (complete IM) with only sialomucin, type II with mixed sialomucin (incomplete IM) and sulfomucin and type III with only sulfomucin (incomplete IM) [8]. Accumulated experiences with human specimens showed the simultaneous expression of different types of mucins in the same metaplastic epithelial cells, suggesting that the metaplastic process represents a gradual phenotypic change [8, 9]. It is unclear whether these three types of IM follow a chronologic sequence. Incomplete IM was considered to be associated with higher gastric cancer risk compared with complete IM. A multicenter study revealed that the incidence rate of GAC was 2.76 and 5.76 per 1000 person-years for complete IM and incomplete IM patients, respectively [10]. In general, patients with IM type III more frequently developed to GAC [11]. Therefore, biomarkers to triage high risk IM patients for GAC will help the surveillance of gastric cancer.

Gastric Cancer Related Gene 213 (GCRG213) was first identified in our lab and was upregulated in human GAC tissue at mRNA level [12]. GCRG213 sequence shared 90% similarity with human long interspersed nucleotide elements (LINE-1, L1) and could be a variant of L1-endonuclease (L1-EN). L1 constitutes a large family of retrotransposable elements, accounting for 17% of the human genome [13]. L1-EN is a part of L1-ORF2p [14, 15]. Our previous study proposed that GCRG213 could be a spliced L1-ORF2 and a variant of L1-EN, since GCRG213 protein (GCRG213p) shares high sequence alignments with L1-EN and possesses conserved residues which are crucial for L1-EN phosphate binding, metal binding and catalytic activity [16]. Overexpression of GCRG213p was reported in both primary GAC and lymph node metastasis [16]. Our preliminary data also showed GCRG213p expression in gastric precancerous lesions, including dysplasia and IM. However, the distribution of GCRG213p expression in the gastric complete and incomplete IM is unclear. In current study, we further investigate the GCRG213p expression in the stepwise system from normal gastric mucosa, IM, dysplasia and adenocarcinoma by immunohistochemistry (IHC). In addition, we compared GCRG213p expression in different subtypes of IM.

## Methods

### Patients

Gastric specimens from patients who underwent gastrectomy for GAC between 2010 and 2013 were retrieved from the Chinese PLA General Hospital, Beijing, China. Paraffin-embedded tumor and paired surrounding gastric mucosa tissues were obtained. Samples of intestinal type adenocarcinoma according to Lauren’s classification, such as papillary and tubular adenocarcinoma, were included, and those with components of diffuse type were excluded. A total of 123 intestinal type GAC cases were collected. Among them, 47 cases had both tumor and para-tumor samples, and 76 cases only had para-tumor IM samples. Besides, three specimens of normal gastric mucosa resected in operation such as Whipple procedure were collected.

### Immunohistochemistry and assessment

Immunohistochemistry for GCRG213p was performed on 4 μm paraffin sections according to procedure described previously [17]. Briefly, after antigen retrieval, monoclonal mouse anti-human GCRG213p antibody, which was produced in our laboratory [18], was added at a dilution of 1:800 and incubated for 2 h at room temperature. The slides were then incubated for 1 h in secondary antibody. An EnVision kit (Dako, Carpinteria, CA, USA) was used to visualize antibody binding, and slides were subsequently counterstained with hematoxylin. A PBS-only staining sample was used as a background control. Positive controls for GCRG213p were represented by sections taken from gastric cancer. Specific immunostaining for GCRG213p was exclusively confined to the cytoplasm. The staining was scored independently and in a blinded manner by two investigators. The inter-observer disagreements were reviewed, followed by a conclusive judgment by both observers. Immunostaining for GCRG213p was scored by staining intensity and the percentage of positively stained cells as described formerly, 0 (absent), 1 (weak), 2 (moderate), and 3 (strong) and 0 (0% positive), 1 (1–30%), 2 (31–60%), and 3(> 60%). The score of intensity and extension was combined and the minimum summed score was 0, and the maximum was 6. An overall score ≥ 3 was deemed a positive GCRG213p expression.

### Histochemical types and classification of IM

Histochemical types of IM was characterized with Alcian blue-periodic acid-Schiff (AB-PAS) and High Iron Diamine-Alcian blue (HID-AB) staining methods. The protocols for AB-PAS and HID-AB staining reported previously were used [19]. All solutions were prepared freshly. Briefly, for AB-PAS staining, after deparaffinization, the paraffin slides were stained with AB staining solution (pH 2.5) for 10 min, washed with distilled water, oxidated with 1.0% periodic acid solution for 10 min, stained with Schiff solution for 10 min and finally washed with distilled water. For HID-AB staining, the paraffin slides were deparaffinized, reacted with high iron diamine solution (pH 1.5–1.6) at room temperature for 24 h, washed with distilled water, stained with AB solution for 10 min, washed with distilled water, stained with 0.5% neutral red solution for 1–2 min and finally washed with distilled water. Based on the morphology and histochemistry findings, IM was classified as three types: type I (complete IM), type II and type III (incomplete IM) [8].

### Statistical analysis

Statistical analyses were performed using SPSS 19.0 (SPSS Inc., Chicago, IL). The differences of GCRG213p expression in GAC, adjacent dysplasia and IM, and the distributions in IM subtypes and in different differentiated degrees of GAC were compared by using Kruskal-Wallis test or Mann -Whitney test and a 2-sided *P* value of 0.05 was used as the criterion for statistical significance.

## Results

### GCRG213p expression pattern in gastric IM subtypes

**Fig. 1:**
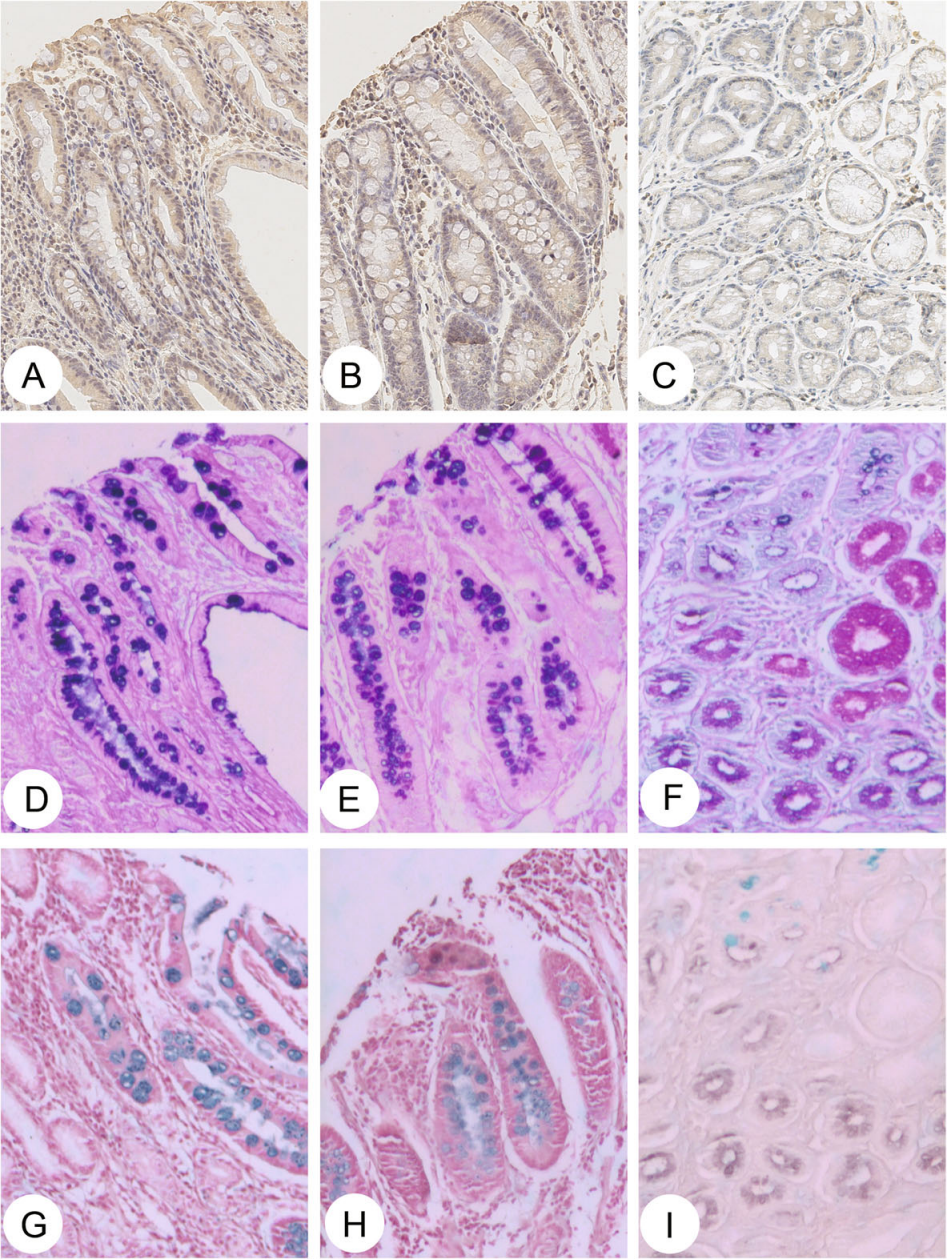
GCRG213p expression and special acid stains in gastric intestinal metaplasia subtypes. Specific yellow-brown staining was identified in cytoplasm of the well-differentiated absorptive cells that have a brush-like border in IM cells. **a**, **b**, **c** with GCRG213p, **a**: type I; **b**: type II; **c**: type III; **d**, **e**, **f** with AB-PAS stains, **d**: type I; **e**: type II; **f**: type III; **g**, **h**, **i** with HID-AB stains, **g**: type I; **h**: type II; **i**: type III

Specimens from 76 cases who only had para-tumor IM samples were used to perform GCRG213p immunohistochemistry, and histochemical staining (Fig. 1). GCRG213p expressed mainly in the cytoplasm of absorptive enterocytes with defined brush borders, but the signal was weak in the enterocytes without brush borders. In the goblet cells, the immunoreactivity was absent. The percentage of GCRG213p in IM type I, type II and type III were 90, 26 and 35%, respectively (Table 1). The expression of GCRG213p among the three IM subtypes differed significantly ((*P* < 0.001), with type I (complete IM) higher than that in type II or type III (incomplete IM) (Table 1). There is no difference between the type II and type III subgroups.

**Table 1:** Expression of GCRG213p in gastric IM subtypes

Subtypes of IM		N	Cases of overall score^a^				PR^b^ (%)	*P* value
			0–2	3	4–5	6		
Complete IM	I	20	2	6	9	3	90.00	*P* < 0.001^c^
Incomplete IM	II	44	28	8	6	2	36.36	*P* < 0.001^d^
	III	12	9	1	2	0	25.00	*P* = 0.001^e^
Total		76	39	15	17	5	48.68	

^a^Overall score calculated as (intensity score) plus (percent cells positive score) as described in methods

^b^PR: Positive rate of GCRG213p expression

^c^Among type I, II and III

^d^Between type I and III

^e^Between type I and II

### GCRG213p expression pattern in human normal gastric mucosa

**Fig. 2:**
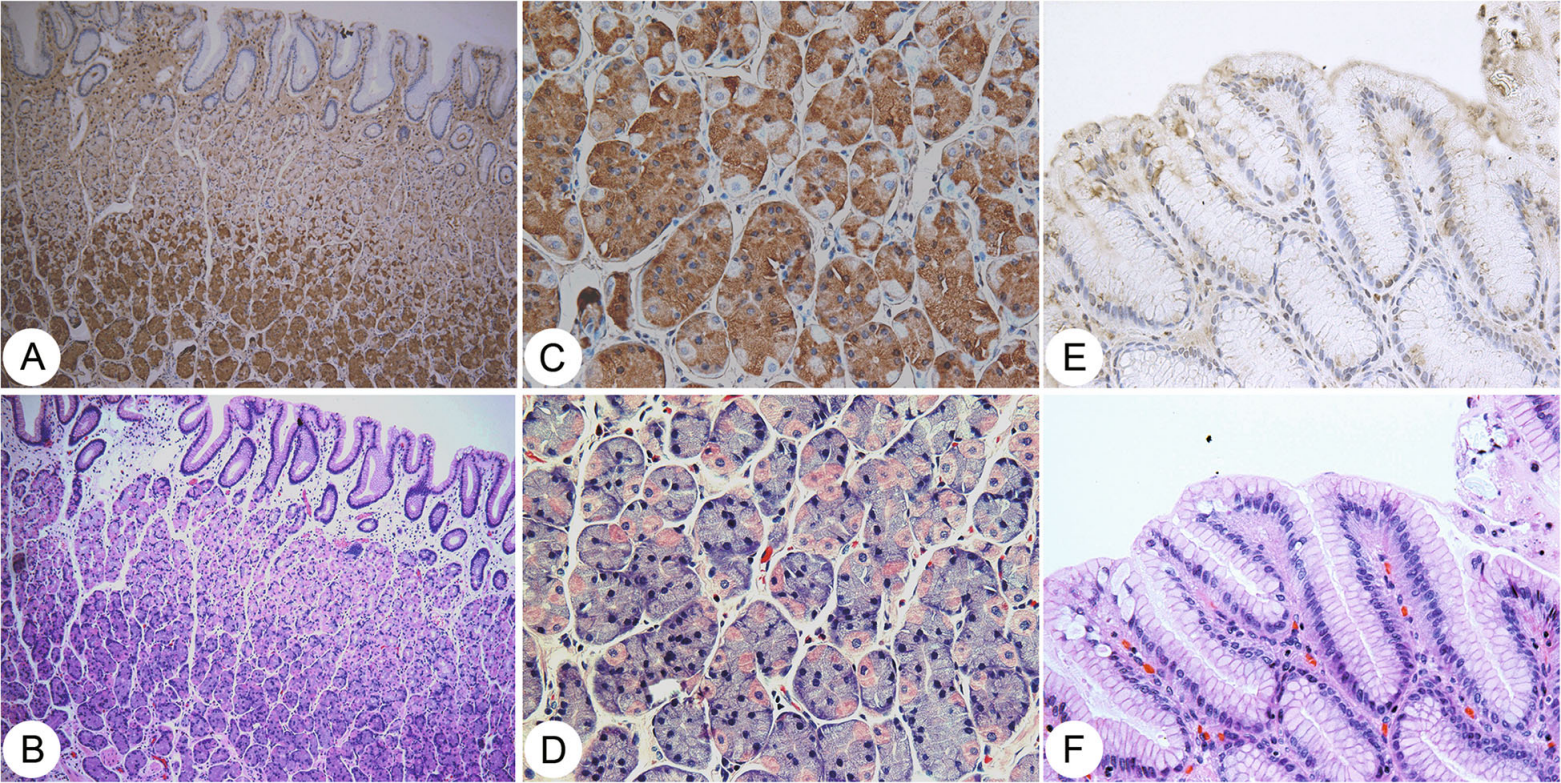
GCRG213p expression in human normal gastric mucosa. Serial sections from the same block of normal gastric fundic mucosa were stained by immunohistochemistry with GCRG213p antibody (the upper row) and H&E (the lower row). **a** and **c**: GCRG213p expression in normal gastric chief cells (**a**: 100×; **c**: 400x, IHC); **e**: GCRG213p was negative in surface faveolar cells (400x). **b**, **d** and **f**: matched gastric mucosa with H&E stain (**b**: 100x; **d**: 400x; **f**: 400x)

GCRG213p was also identified in normal gastric mucosa samples with yellow-brown signal at the bottom of gastric glands where the gastric chief cells located. Other cells in the gastric mucosa, such as parietal cells, surface foveolar cells and pylori glands in gastric antrum were negative (Fig. 2).

### GCRG213p expression in gastric IM, dysplasia and gastric adenocarcinoma

**Fig. 3:**
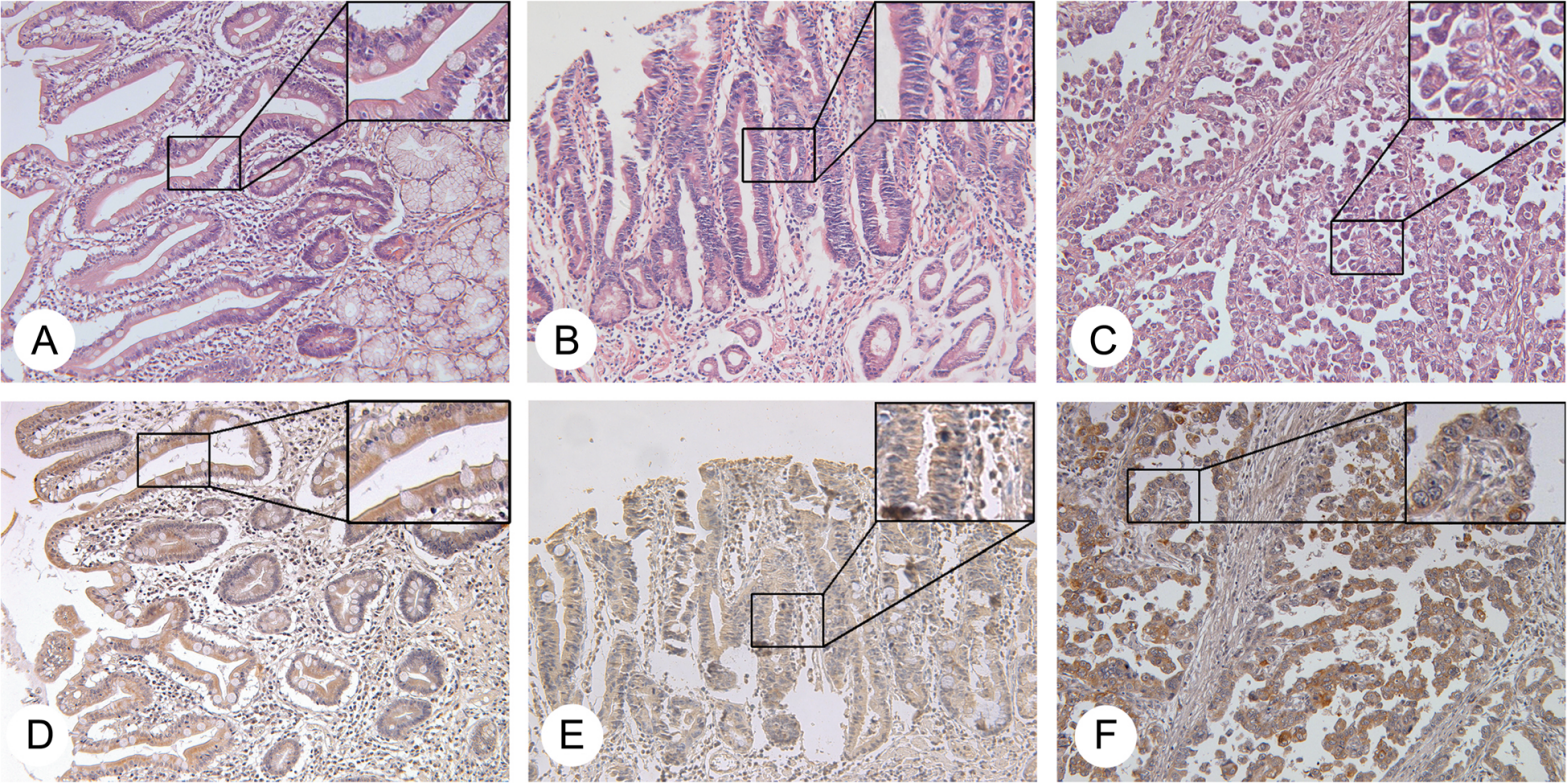
GCRG213p expression in IM, gastric dysplasia and adenocarcinoma. **a**-**c**, **h** & **e** staining images in gastric IM (**a**), dysplasia (**b**) and adenocarcinoma (**c**). **d**-**f**, representative images of GCRG213p immunostaining in tissue samples from gastric IM (**d**), dysplasia (**e**) and adenocarcinoma (**f**). (Original magnification 200×, and 400× in block frame)

Tissues from 47 patients who had both tumor and para-tumor samples were studied with GCRG213p immunohistochemistry. Specific yellow-brown immunostaining for GCRG213p were found in cytoplasm of the positive cells (Fig. 3). GCRG213p was extensively expressed in IM, dysplasia and GAC at 48.9, 57.1 and 55.3%, respectively (Table 2). There was no significantly difference of GCRG213p expression among IM, dysplasia and GAC (*P* = 0.956), but a significant difference of GCRG213p expression between well-to-moderately-differentiated and poorly-differentiated GAC was noticed (*P* = 0.036).

**Table 2:** GCRG213p expression in intestinal metaplasia (IM), dysplasia and gastric adenocarcinoma

Lesions	N	Cases of overall score^a^				PR^b^ (%)	*P* value
		0–2	3	4–5	6		
IM	47	24	7	6	10	48.94	
Dysplasia	7	3	1	2	1	57.14	*P* = 0.956^c^
Gastric adenocarcinoma	47	21	11	10	5	55.32	
Well-to-moderately-differentiated	14	3	3	4	4	78.57	*P* = 0.036^d^
Poorly-differentiated	33	18	6	4	5	45.45	

^a^Overall score calculated as (intensity score) plus (percent cells positive score) as described in methods

^b^PR: Positive rate of GCRG213p expression

^c^Among gastric IM, dysplasia and adenocarcinoma

^d^Between well-to-moderately-differentiated and poorly-differentiated adenocarcinoma

## Discussion

It is now clear that 2 types of mucous cell metaplasia develop in the atrophic human stomach and represent putative preneoplastic lesions: goblet cell IM and spasmolytic polypeptide–expressing metaplasia (SPEM, also known as *pseudopyloric metaplasia*) [20, 21] . IM is defined as the replacement of glandular and/or foveolar epithelia by intestinal epithelia and SPEM as the trans-differentiation of chief cells with TFF2 expression in gastric mucosa [22]. The true identity of original cells in gastric IM remains to be established, but two competing models are advocated [23]. Some studies propose that the cell of origin for IM resides in the gastric isthmus [24]. However, other researchers have observed some cells that expressed both TFF2, a SPEM marker, and MUC2, a goblet marker. In the fundus of Helicobacter pylori-infected Mongolian gerbils, goblet cells in SPEM glands were observed in the later stages of infection [21, 25]. Therefore, IM may arise from SPEM [26] or perhaps directly from chief cells [27]. Indeed, Troy+ chief cells are reported to produce all epithelial lineages present in the corpus in vivo [28]. One recent study even showed that SPEM can arise by direct reprogramming of existing chief cells, without contribution from gastric stem cells [29], which implies there exists genetic continuum between differentiated chief cells and IM. In this study, we found that GCRG213p positive presented exclusively in chief cells and in a high percentage of IM glands. Thus, it is meaningful to speculate that GCRG213p may be related with the cell differentiation from the chief cell to IM. The mechanism of GCRG213p on the IM from the chief cells is worth to be further studied.

Intestinal metaplasia in the stomach can progress to low-grade dysplasia and high-grade dysplasia and culminate in GAC [30]. Because of the large surface area of gastric mucosa and the lack of targeted sampling strategy, the true frequency of IM progression to adenocarcinoma in the stomach is difficult to discern. The relative risks of GAC were reported from 4- to 11-fold higher for the presence of incomplete type in comparison to complete type [31]. A significant association has been documented between incomplete IM and extensive/multifocal IM [32]. Extensive gastric IM has been recommended as a high-risk marker for gastric cancer, according to international recommendations and guidelines [33, 34]. Therefore, apart from a routine combination of morphology (Haemotoxylin and Eosin, H&E staining) and mucins staining (AB-PAS and HID-AB), GCRG213p, whose expression was significantly different between complete and incomplete IM, might provide a good merit on evaluating sub-type of IM.

After analyzing gene expression and molecular processes involved in IM subtypes, the incomplete IM shows a higher number of up-regulated differentially expressed genes and molecular processes than complete IM, which is in agreement with its higher risk of progression to GC [35]. In fact, complete IM is merely a weak risk factor for gastric cancer, and it may even suppress cancer development [10, 36]. Our study showed that the expression of GCRG213p in IM type I (complete IM) was significantly higher than in IM type II and type III (incomplete IM), which implied that GCRG213p may play a role to decrease the possibility of developing from IM to dysplasia or adenocarcinoma. L1-EN is believed to produce the nicking of genomic deoxyribonucleic acid (DNA). Thus, even simply upregulation of L1-EN could promote the formation of additional double strand breaks of DNA, which resulted in cell cycle arrest, apoptosis or senescence. A correlation between L1 expression/retrotransposition and induction of apoptosis was observed in breast cancer cells, which implies that DNA nicks created by L1 expression and retrotransposition are sensed as a DNA damaging event, which leads to apoptosis [37]. Therefore, GCRG213p, a L1-EN variant, may have a similar function to induce cell cycle arrest, apoptosis or senescence in IM, which could explain the complete IM has a low risk for developing to GAC.

## Conclusions

The expression of GCRG213p in complete IM is significantly higher than in incomplete IM, which implies that GCRG213 may play a protective role on the developing from IM to adenocarcinoma. This differential expression pattern also reminds a possible role of GCRG213p as a biomarker of IM sub-typing. GCRG213p is exclusively expressed in chief cells, which suggests that GCRG213p may be associated with the cell differentiation from the chief cells to IM.

## Data Availability

The data used and/or analyzed during the current study are available from the corresponding author on reasonable request.

## Abbreviations

AB-PAS: Alcian blue-periodic acid-Schiff staining
DNA: Deoxyribonucleic acid
GAC: Gastric adenocarcinoma
GCRG213p: Gastric cancer related gene 213 protein
H & E: Haemotoxylin and Eosin
HID-AB: High Iron Diamine-Alcian blue staining
IHC: Immunohistochemistry
IM: Intestinal metaplasia
L1-EN: L1-endonuclease
LINE-1, L1: Long interspersed nucleotide elements-1
SPEM: Spasmolytic polypeptide–expressing metaplasia

## References

[1] Disease GBD, Injury I, Prevalence C (2017). Global, regional, and national incidence, prevalence, and years lived with disability for 328 diseases and injuries for 195 countries, 1990-2016: a systematic analysis for the global burden of Disease study 2016. Lancet.

[2] Correa P (1992). Human gastric carcinogenesis: a multistep and multifactorial process--first American Cancer Society award lecture on Cancer epidemiology and prevention. Cancer Res.

[3] Zivny J, Wang TC, Yantiss R, Kim KH, Houghton J (2003). Role of therapy or monitoring in preventing progression to gastric cancer. J Clin Gastroenterol.

[4] Shichijo S, Hirata Y, Niikura R, Hayakawa Y, Yamada A, Ushiku T, Fukayama M, Koike K (2016). Histologic intestinal metaplasia and endoscopic atrophy are predictors of gastric cancer development after helicobacter pylori eradication. Gastrointest Endosc.

[5] Marques-Lespier JM, Gonzalez-Pons M, Cruz-Correa M (2016). Current perspectives on gastric Cancer. Gastroenterol Clin N Am.

[6] de Vries AC, van Grieken NC, Looman CW, Casparie MK, de Vries E, Meijer GA, Kuipers EJ (2008). Gastric cancer risk in patients with premalignant gastric lesions: a nationwide cohort study in the Netherlands. Gastroenterology.

[7] Song H, Ekheden IG, Zheng Z, Ericsson J, Nyren O, Ye W (2015). Incidence of gastric cancer among patients with gastric precancerous lesions: observational cohort study in a low risk Western population. BMJ.

[8] Filipe MI, Munoz N, Matko I, Kato I, Pompe-Kirn V, Jutersek A, Teuchmann S, Benz M, Prijon T (1994). Intestinal metaplasia types and the risk of gastric cancer: a cohort study in Slovenia. Int J Cancer.

[9] Piazuelo MB, Haque S, Delgado A, Du JX, Rodriguez F, Correa P (2003). Phenotypic differences between esophageal and gastric intestinal metaplasia. Mod Pathol.

[10] Gonzalez CA, Sanz-Anquela JM, Companioni O, Bonet C, Berdasco M, Lopez C, Mendoza J, Martin-Arranz MD, Rey E, Poves E (2016). Incomplete type of intestinal metaplasia has the highest risk to progress to gastric cancer: results of the Spanish follow-up multicenter study. J Gastroenterol Hepatol.

[11] Rokkas T, Filipe MI, Sladen GE (1991). Detection of an increased incidence of early gastric cancer in patients with intestinal metaplasia type III who are closely followed up. Gut.

[12] Wang GS, Wang MW, Wu BY, Liu XB, You WD, Yang XY (2003). A gene encoding an apurinic/apyrimidinic endonuclease-like protein is up-regulated in human gastric cancer. World J Gastroenterol.

[13] Lander ES, Linton LM, Birren B, Nusbaum C, Zody MC, Baldwin J, Devon K, Dewar K, Doyle M, FitzHugh W (2001). Initial sequencing and analysis of the human genome. Nature.

[14] Feng Q, Moran JV, Kazazian HH, Boeke JD (1996). Human L1 retrotransposon encodes a conserved endonuclease required for retrotransposition. Cell.

[15] Mathias SL, Scott AF, Kazazian HH, Boeke JD, Gabriel A (1991). Reverse transcriptase encoded by a human transposable element. Science.

[16] Wang G, Gao J, Huang H, Tian Y, Xue L, Wang W, You W, Lian H, Duan X, Wu B (2013). Expression of a LINE-1 endonuclease variant in gastric cancer: its association with clinicopathological parameters. BMC Cancer.

[17] Going JJ, Stuart RC, Downie M, Fletcher-Monaghan AJ, Keith WN (2002). ‘Senescence-associated’ beta-galactosidase activity in the upper gastrointestinal tract. J Pathol.

[18] Wu YQ, Wu BY, Wang GS, You WD, Wang WH, Wang MW (2012). Preparation and identification of monoclonal antibody against human GCRG213. Xi Bao Yu Fen Zi Mian Yi Xue Za Zhi.

[19] Jass JR, Filipe MI (1981). The mucin profiles of normal gastric mucosa, intestinal metaplasia and its variants and gastric carcinoma. Histochem J.

[20] Schmidt PH, Lee JR, Joshi V, Playford RJ, Poulsom R, Wright NA, Goldenring JR (1999). Identification of a metaplastic cell lineage associated with human gastric adenocarcinoma. Lab Investig.

[21] Choi E, Hendley AM, Bailey JM, Leach SD, Goldenring JR (2016). Expression of activated Ras in gastric chief cells of mice leads to the full Spectrum of metaplastic lineage transitions. Gastroenterology.

[22] Mills JC, Shivdasani RA (2011). Gastric epithelial stem cells. Gastroenterology.

[23] Giroux V, Rustgi AK (2017). Metaplasia: tissue injury adaptation and a precursor to the dysplasia-cancer sequence. Nat Rev Cancer.

[24] Hayakawa Y, Fox JG, Wang TC (2017). Isthmus stem cells are the origins of metaplasia in the gastric Corpus. Cell Mol Gastroenterol Hepatol.

[25] Yoshizawa N, Takenaka Y, Yamaguchi H, Tetsuya T, Tanaka H, Tatematsu M, Nomura S, Goldenring JR, Kaminishi M (2007). Emergence of spasmolytic polypeptide-expressing metaplasia in Mongolian gerbils infected with helicobacter pylori. Lab Investig.

[26] Goldenring JR, Nam KT, Mills JC (2011). The origin of pre-neoplastic metaplasia in the stomach: chief cells emerge from the mist. Exp Cell Res.

[27] Mills JC, Goldenring JR (2017). Metaplasia in the stomach arises from gastric chief cells. Cell Mol Gastroenterol Hepatol.

[28] Stange DE, Koo BK, Huch M, Sibbel G, Basak O, Lyubimova A, Kujala P, Bartfeld S, Koster J, Geahlen JH (2013). Differentiated Troy+ chief cells act as reserve stem cells to generate all lineages of the stomach epithelium. Cell.

[29] Radyk MD, Burclaff J, Willet SG, Mills JC (2018). Metaplastic cells in the stomach Arise, independently of stem cells, via dedifferentiation or Transdifferentiation of chief cells. Gastroenterology.

[30] Amieva M, Peek RM (2016). Pathobiology of helicobacter pylori-induced gastric Cancer. Gastroenterology.

[31] Gonzalez CA, Sanz-Anquela JM, Gisbert JP, Correa P (2013). Utility of subtyping intestinal metaplasia as marker of gastric cancer risk. A review of the evidence. Int J Cancer.

[32] Quach DT, Le HM, Hiyama T, Nguyen OT, Nguyen TS, Uemura N (2013). Relationship between endoscopic and histologic gastric atrophy and intestinal metaplasia. Helicobacter.

[33] Correa P, Piazuelo MB, Wilson KT (2010). Pathology of gastric intestinal metaplasia: clinical implications. Am J Gastroenterol.

[34] Dinis-Ribeiro M, Areia M, de Vries AC, Marcos-Pinto R, Monteiro-Soares M, O'Connor A, Pereira C, Pimentel-Nunes P, Correia R, Ensari A (2012). Management of precancerous conditions and lesions in the stomach (MAPS): guideline from the European Society of Gastrointestinal Endoscopy (ESGE), European helicobacter study group (EHSG), European Society of Pathology (ESP), and the Sociedade Portuguesa de Endoscopia Digestiva (SPED). Endoscopy.

[35] Companioni O, Sanz-Anquela JM, Pardo ML, Puigdecanet E, Nonell L, Garcia N, Parra Blanco V, Lopez C, Andreu V, Cuatrecasas M (2017). Gene expression study and pathway analysis of histological subtypes of intestinal metaplasia that progress to gastric cancer. PLoS One.

[36] Pittayanon R, Rerknimitr R, Klaikaew N, Sanpavat A, Chaithongrat S, Mahachai V, Kullavanijaya P, Barkun A (2017). The risk of gastric cancer in patients with gastric intestinal metaplasia in 5-year follow-up. Aliment Pharmacol Ther.

[37] Belgnaoui S Mehdi, Gosden Roger G, Semmes O John, Haoudi Abdelali (2006). Cancer Cell International.

